# Pre-ablation rate control therapy and direct-to-catheter ablation in patients with persistent atrial fibrillation: A DECAAF II subanalysis

**DOI:** 10.1007/s10840-025-02218-1

**Published:** 2026-01-06

**Authors:** Radia Ksayer, Hadi Younes, Eli Tsakiris, Bella Gonzalez Perez, Yishi Jia, Alina Upreti, Ghassan Bidaoui, Christian Massad, Amitabh C. Pandey, Han Feng, Nassir F. Marrouche

**Affiliations:** Tulane Research Innovation for Arrhythmia Discovery, New Orleans, 1430 Tulane Avenue, LA 70112 USA

**Keywords:** AFIB, Ablation, Rate control, AF recurrence, Beta blocker, CCB

## Abstract

**Background:**

For patients with persistent atrial fibrillation (PersAF), initial treatment strategies often involve rate or rhythm control before offering ablation. This study compared two approaches: (1) catheter ablation without prior medication (referred to as direct-to-catheter ablation, DTCA) and (2) catheter ablation after initial rate control with either diltiazem or metoprolol.

**Objective:**

This study included two independentanalyses of patients with persistent atrial fibrillation (PersAF) undergoing catheter ablation. Aimed at evaluating the potential impact of pre-ablation rate control medications including beta blockers and calcium channel blockers on post-ablation outcomes. Comparison 1: DTCA without prior beta-blocker use (*n* = 209) vs. metoprolol use prior to ablation (*n* = 260). Comparison 2: DTCA without prior calcium channel blocker use (*n* = 639) vs. diltiazem use prior to ablation (*n* = 55).

**Methods:**

Patients were followed for 18 months to evaluate primary outcome: recurrence of atrial fibrillation (AF) and secondary outcomes: Pre-ablation and Post-ablation left atrial percent fibrosis as seen on LGE MRI and Quality of life (QoL), measured with the SF-36 questionnaire. The Wilcoxon tests were conducted to compare the QoL and fibrosis among groups. Time to recurrence among the groups post ablation was assessed via Kaplan-Meier curves. Multivariable Cox models were developed to adjust for other confounders of AF recurrence.

**Results:**

In the beta-blocker analysis (*n* = 469), no significant difference in AF recurrence was observed between patients without prior beta-blocker use (DTCA group) and those treated with metoprolol (Kaplan-Meier, *p* > 0.05). Similarly, in the calcium channel blocker analysis (*n* = 694), no difference in recurrence was found between the DTCA group and those with prior diltiazem use (*p* > 0.05). Multivariable Cox models confirmed that neither metoprolol (*p* = 0.44) nor diltiazem (*p* = 0.34) independently predicted AF recurrence. Additionally, no significant differences were found in imaging metrics or QoL between the groups in either comparison (all *p* > 0.05).

**Conclusion:**

Prior treatment with diltiazem or metoprolol before ablation of PersAF did not show additional benefits in reducing patient outcomes such as AF recurrence, fibrosis, or improving QoL.

**Graphical Abstract:**

**Figure:**
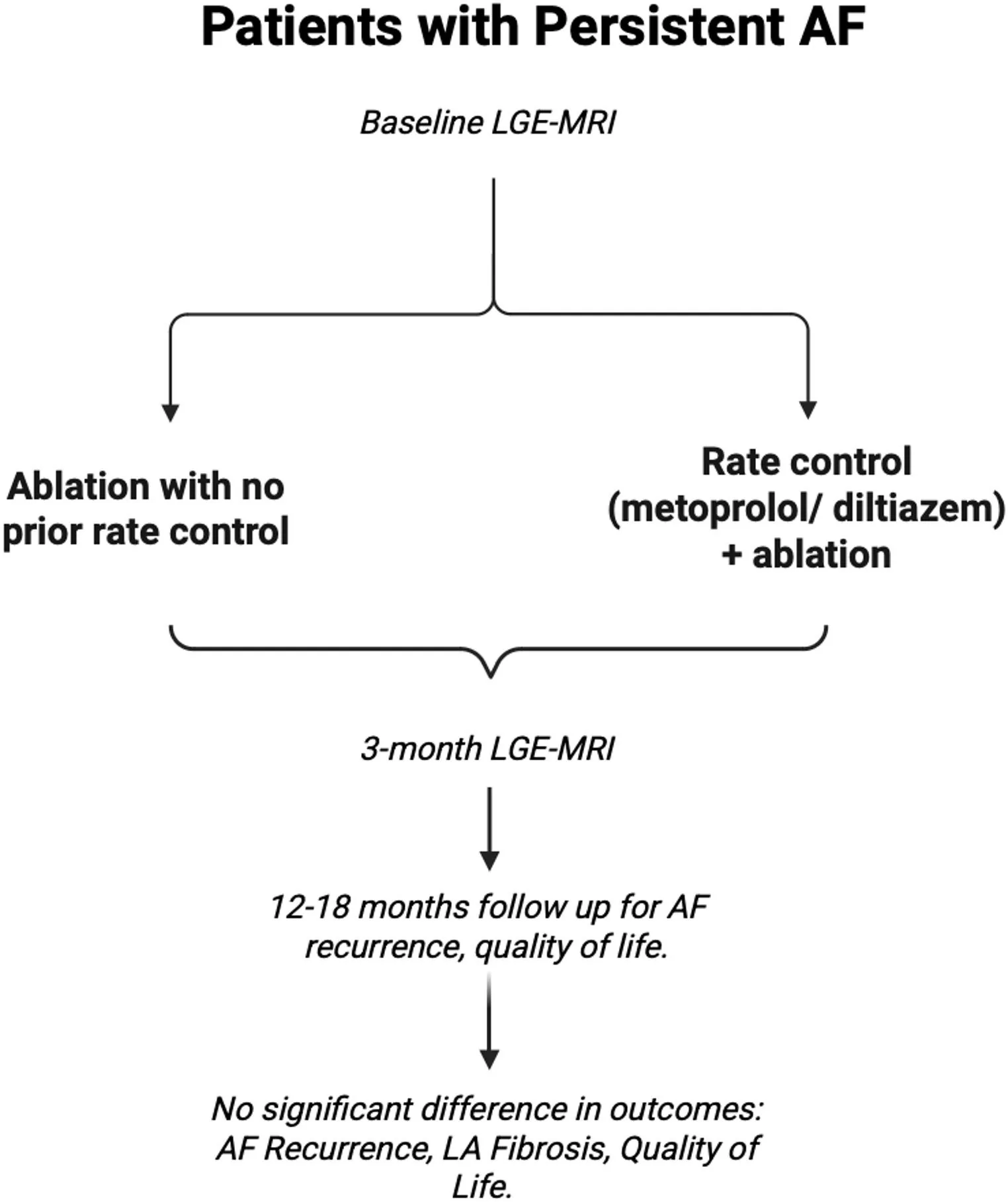

##  Introduction

Atrial fibrillation (AF) is the most common arrhythmia in the world, affecting nearly 1 in every 3–5 individuals worldwide [1]. While early detection and treatment improve outcomes, AF remains one of the most significant risk factors for cardiovascular events and increases the risk of ischemic stroke 5-fold. Medications acting on the atrio-ventricular (AV) node have historically served as first-line treatment for AF, with the 2023 AHA/ACC guidelines endorsing beta-blockers or nondihydropyridine calcium channel blockers as first-line rate control agents, with selection guided by underlying left ventricular function [2]. Utilization of these specific agents is termed rate control due to their function in decreasing ventricular rate independent of atrial rate. Alternatively, a second pharmacologic approach called rhythm control utilizes antiarrhythmic drugs (AADs) such as amiodarone, sotalol, and flecainide that modulate cardiac ion channels (primarily sodium and potassium) to restore or maintain sinus rhythm. While considered interchangeable in efficacy for treatment of AF, rhythm control agents have been associated with more morbid side effects, and rate control agents demonstrated better symptomatic outcomes, particularly in older AF patients [2]. 

Minimally invasive catheter ablation is a proven safe and effective treatment for patients with a spectrum of AF phenotypes. The AHA/ACC 2023 guidelines endorse catheter ablation as a class 1 indication in young and healthy patients with paroxysmal atrial fibrillation (PAF) [2]. Additionally, the recent ESC 2024 guidelines expanded the recommendation of early ablation as a first-line treatment for any patient with PAF [3]. This recommendation was based on studies demonstrating that early catheter ablation may benefit individuals with less advanced AF disease [4]. A direct-to-catheter approach (DTCA) describes treating AF with catheter ablation without a preceding trial of rate or rhythm control. In patients with PersAF, previous post hoc analysis of the DECAAF II trial has shown that rhythm control before catheter ablation did not improve outcomes [5]. 

Pre-ablation rate control might theoretically attenuate tachycardia-induced ventricular dysfunction, neuro-hormonal activation, and atrial stretch that contribute to progressive atrial remodeling. However, since AV-nodal blockade does not suppress atrial fibrillation itself, atrial remodeling may continue despite ventricular rate control. Therefore, it remains unclear whether rate control before ablation confers any measurable benefit in patients with persistent AF.

We performed a retrospective sub-analysis of DECAAF II comparing outcomes among patients who underwent catheter ablation with versus without prior rate-control therapy using metoprolol or diltiazem, the most commonly used beta-blocker and calcium-channel blocker for AF, respectively.

## Methods

### DECAAF II design

DECAAF II is a randomized multi-center clinical trial that included 843 patients with PersAF who were randomly assigned to pulmonary vein isolation (PVI) plus MRI-guided atrial fibrosis ablation (421 patients) or PVI alone (422 patients). The primary tools used in these procedures were radiofrequency ablation (RFA) and cryoablation catheters. For those in the fibrosis-guided group, fibrosis was essentially targetted using processed LGE-MRI as previously described in the DECAAF II protocol. In contrast, the PVI-only group focused solely on the electrical isolation of all pulmonary veins, per the Heart Rhythm Society Consensus Statement. Additional ablation measures were available to address recurrent arrhythmias, at the operator’s discretion, if a normal sinus rhythm was not restored post-PVI, even after cardioversion.

Patients were followed over 12–18 months using a mobile single-lead ECG monitoring device to observe AF recurrence and burden. Patients were instructed to send one ECG recording from the device daily during the study period through a smartphone application. Patients underwent a delayed-enhancement MRI using the Merisight delayed-enhancement MRI protocol (MARREK Inc.) within 30 days before the ablation procedure and 3 months post-ablation. Quality of life was assessed at baseline and 12 months using the standardized Short Form Health Survey (SF-36). The complete methodology is further described in the DECAAF II protocol [6]. 

### Sub-analysis design

All patients in the DECAAF II study with complete data were considered for analysis. Two separate analyses were performed to evaluate the usage of metoprolol or diltiazem before catheter ablation. The first comparison evaluates the use of metoprolol; patients undergoing DTCA without a history of beta blocker use (including but not limited to metoprolol, carvedilol, or propranolol) were compared to patients with a history of metoprolol use before ablation. The second comparison evaluates diltiazem use; patients undergoing DTCA without a history of CCB use (including but not limited to diltiazem, verapamil, amlodipine, or nifedipine) were compared to patients with a history of diltiazem use before ablation.

As described in DECAAF II, the primary endpoint of the study was the first confirmed recurrence of atrial arrhythmia (including AF, atrial flutter, or atrial tachycardia) lasting for at least 30 s after the 90-day blanking period, demonstrated by at least two consecutive 1-lead smartphone ECG device tracings, one positive reading on a clinical 12-lead ECG tracing, ambulatory monitor, or if the patient underwent repeat ablation. The daily smartphone ECGs were used for assessing atrial arrhythmia recurrence. Clinical and ambulatory ECGs were also used for detecting AF recurrence when deemed necess. A core laboratory at the University of Washington adjudicated the ECG findings. This sub-analysis utilized the 36-item Short Form Survey (SF-36) score as the primary subjective outcome metric to evaluate the quality of life at baseline and 12 months. Baseline MRI results were utilized to quantify left atrial (LA) fibrosis and LA volume in all patients at baseline and the 3-month follow-up. LA Volume was indexed to body surface area. Patients were followed for 12–18 months.

### Statistical analysis

Demographics and common comorbidities were compared between each group pairing within their respective analysis (metoprolol group vs. no beta-blocker group, and diltiazem group vs. no CCB group). Normality was assessed using the Shapiro-Wilk test for continuous variables, with Wilcoxon and t-tests conducted accordingly, depending on normality. Continuous variables are presented as median with standard deviation in parentheses. Categorical variables were tested with Chi-square tests and presented with counts and percentages of the specific study group. The Kaplan-Meier curve was used to evaluate the univariate effect of the independent variable, use of rate control agent before ablation, on the primary outcome of AF recurrence after ablation. Corresponding p-values are generated using the log-rank test. Two separate multivariable Cox models, one for each analysis, assessed the independent effects of clinically relevant variables. They were selected based on individual significance in t-tests assessing their impact on the primary outcomes. All statistical analysis was performed using R (version 4.3.0) software, with a 2-sided significance level of 0.05.

## Results

### Baseline characteristics

A total of 760 patients within DECAAF II were considered for analysis, with 199 excluded for insufficient LGE MRI data. The mean age of patients was 62.0 ± 9.0 years, and 79.5% were males. 260 patients taking metoprolol before catheter ablation were identified and compared to 209 patients not taking any beta blocker before ablation. 55 patients taking diltiazem before ablation were identified and compared to 639 patients not taking any CCB before ablation. Baseline characteristics of all groups, in their respective comparison pairings, are shown in Tables 1 and 2.

**Table 1:** Demographics (Direct-to-Catheter vs. Pre-ablation Metoprolol groups)

Variable	Direct-to-Catheter without prior Beta Blocker (*N* = 209)	Pre-ablation Metoprolol (*N* = 260)	*p*-value
Age, years	62.0 ± 8.9	62.4 ± 9.5	0.54
Sex (male)	164 (78.5%)	199 (76.5%)	0.62
BMI	30.8 ± 5.6	32.4 ± 6.9	0.006
Left atrial volume (mL)	126.6 ± 37.6	130.0 ± 40.9	0.016
Baseline fibrosis (%)	18.6 ± 7.4	19.1 ± 7.3	0.52
Diabetes Mellitus	15 (7.2%)	37 (14.2%)	0.016
Congestive Heart Failure	24 (11.5%)	54 (20.8%)	0.007
Hypertension	105 (50.2%)	159 (61.2%)	0.018
Coronary Artery Disease	22 (10.5%)	32 (12.3%)	0.55
Vascular Disease	14 (6.7%)	33 (12.7%)	0.032
Stroke history	16 (7.7%)	31 (11.9%)	0.13
Aldosterone Inhibitor Use	14 (6.7%)	16 (6.2%)	0.81
*Randomized treatment group*:			0.40
Conventional PVI	111 (53.1%)	128 (49.2%)	
PVI + Fibrosis-guided Ablation	98 (46.9%)	132 (50.8%)

**Table 2:** Demographics (Direct-to-catheter vs. Pre-ablation diltiazem groups)

Variable	Direct-to-Catheter without prior Calcium Channel Blocker (*N* = 639)	Pre-ablation diltiazem (*N* = 55)	*p*-value
Age, years	61.5 ± 9.2	64.4 ± 8.1	0.048
Sex (male)	513 (80.3%)	41 (74.5%)	0.31
BMI	31.0 ± 6.1	34.2 ± 9.0	< 0.001
Left atrial volume (mL)	129.5 ± 41.4	137.4 ± 37.5	0.092
Baseline fibrosis (%)	18.9 ± 7.6	18.7 ± 7.6	0.78
Diabetes Mellitus	56 (8.8%)	9 (16.4%)	0.06
Congestive Heart Failure	122 (19.1%)	15 (27.3%)	0.14
Hypertension	331 (51.8%)	41 (74.5%)	< 0.001
Coronary Artery Disease	73 (11.4%)	8 (14.5%)	0.49
Vascular Disease	62 (9.7%)	2 (3.6%)	0.14
Stroke history	52 (8.1%)	5 (9.1%)	0.81
Aldosterone Inhibitor Use	54 (8.5%)	1 (1.8%)	0.08
*Randomized treatment group*:			0.50
Conventional PVI	318 (49.8%)	30 (54.5%)	
PVI + Fibrosis-guided Ablation	321 (50.2%)	25 (45.5%)	

### Use of metoprolol before ablation

Patients taking pre-ablation metoprolol group had higher BMI (32.4 vs. 30.8, *p* = 0.006) higher rates of congestive heart failure (20.8% vs. 11.5%, *p* = 0.007), vascular disease (12.7% vs. 6.7%, *P* = 0.032), diabetes mellitus (14.2% vs. 7.2%, *p* = 0.016) and hypertension (61.2% vs. 50.2%, *p* = 0.018) as compared to those undergoing DTCA without Beta-blocker. Kaplan Meier survival analysis showed no significant difference in AF recurrence following ablation (HR = 1.12, 95%CI (0.84, 1.49), *p* = 0.44) between these two groups (Fig. 1). Mean quality of life as measured by SF36 scores was also similar between both groups at 3months (78.6 ± 19.0 vs. 79.4 ± 17.2, *p* = 0.88) (Fig. 2). Left atrial volume index (LAVI) decrease at 12 months after ablation was also similar between groups (11.4 mm^3^ vs. 10.4 mm^3^, *p* = 0.298). Additionally, on LGE-MRI there were no significant differences in baseline fibrosis (19.1% ± 7.3% vs. 18.6% ± 7.4%, *p* = 0.52), fibrosis post-ablation (15.0% ± 6.5% vs. 14.1% ± 5.9%, *p* = 0.27) = 0.65) (Table 3).

**Table 3:** Direct-to-catheter vs. pre-ablation metoprolol: fibrosis

Variable	DTCA (No BB) (*N* = 209)	Pre-ablation metoprolol (*N* = 260)	Total (*N* = 469)	*p*-value
Fibrosis				
Baseline fibrosis (%)	18.6 ± 7.4	19.1 ± 7.3	18.9 ± 7.3	0.52
Post ablation fibrosis (%)	14.1 ± 5.9	15.0 ± 6.5	14.6 ± 6.2	0.27

**Fig. 1:**
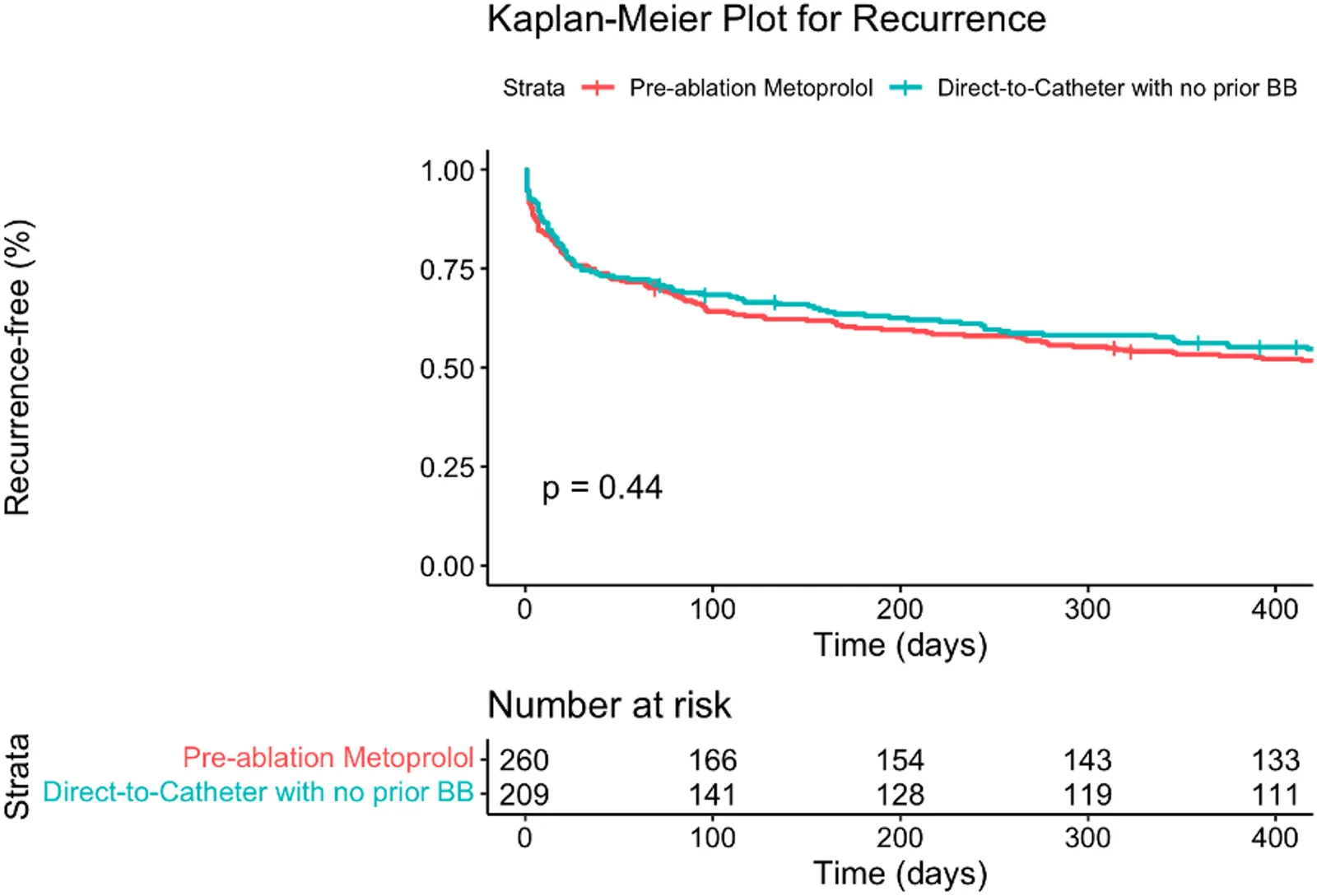
Comparison of AF Recurrence Following Catheter Ablation (Pre-ablation Metoprolol vs. Direct to Catheter Ablation with no Prior Beta Blocker). A Kaplan Meier curve comparing the risk of atrial fibrillation recurrence, in days, following catheter ablation between pre-ablation metoprolol vs direct-to-catheter ablation with no prior beta blocker. No significant difference was found in recurrence between the two groups (p = 0.44)

**Fig. 2:**
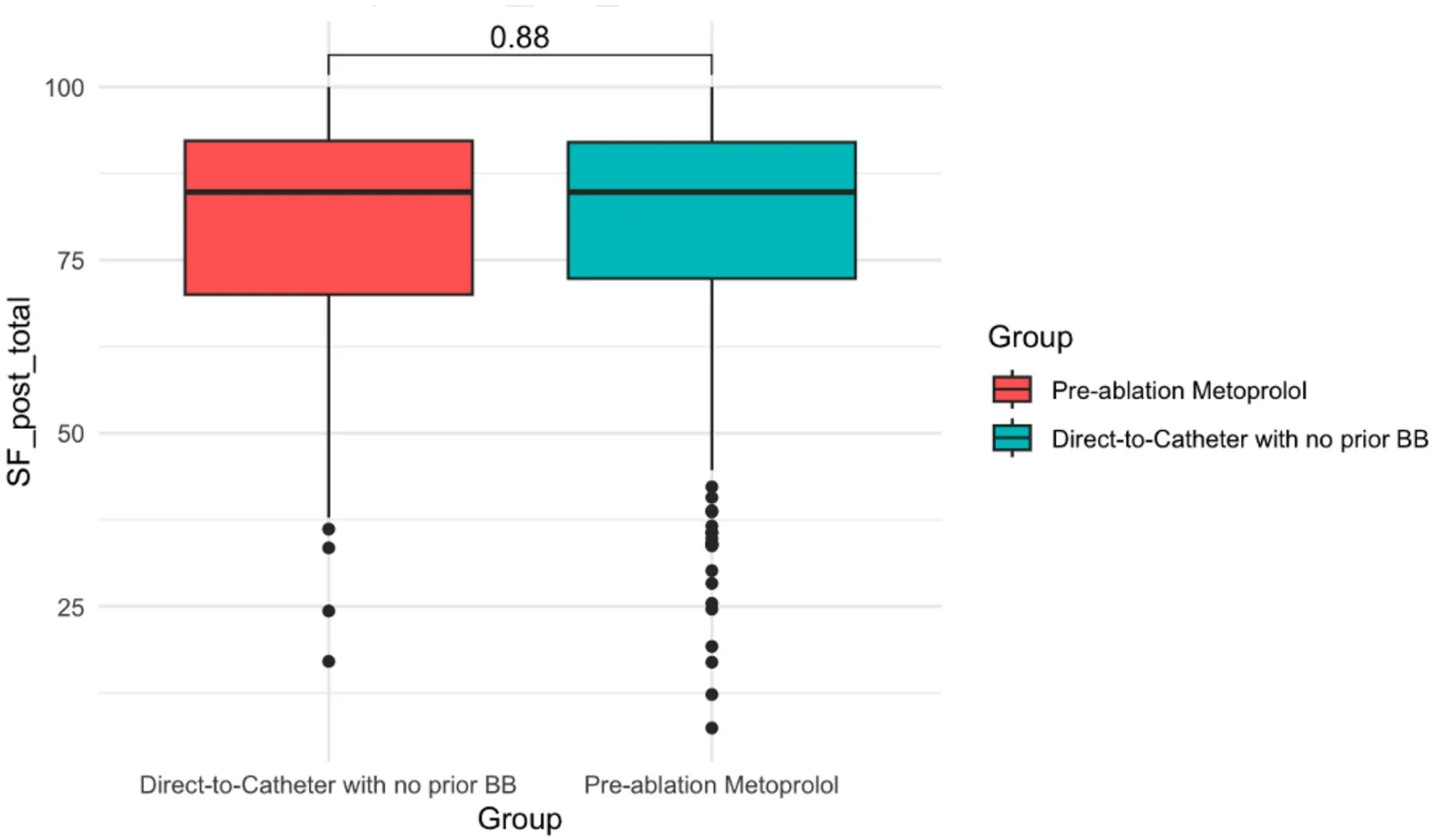
Comparison of Quality-of-Life Following Ablation (Pre-ablation Metoprolol vs Direct to Catheter Ablation with no Prior Beta Blocker). A box plot demonstrating similar quality of life, as measured by the SF 36 questionnaire, between the pre-ablation metoprolol group and the direct-to-catheter ablation without prior beta blocker group (p=.88)

### Use of diltiazem before ablation

Patients taking pre-ablation diltiazem were slightly older (64.4 vs. 61.5, *p* = 0.048), had higher BMI (34.3 vs. 30.9, *p* < 0.001), and had higher rates of hypertension (74.5% vs. 51.8%, *p* < 0.001) as compared to those undergoing DTCA without CCB. Kaplan Meier survival analysis showed no significant difference in AF recurrence following ablation (HR = 1.12, 95%CI (0.75, 1.67), *p* = 0.57) between patients taking diltiazem before ablation and those not taking any CCB before ablation (Fig. 3). Mean quality of life scores were similar between both groups (77.5 ± 16.7 vs. 78.9 ± 18.4, *p* = 0.31) (Fig. 4). LAVI was also similar at 3 months post-ablation. Additionally, no significant differences were seen on LGE MRI for baseline fibrosis (18.7% ± 7.6% vs. 18.9% ± 7.6%, *p* = 0.78), fibrosis post ablation (14.3% ± 7.0% vs. 14.7% ± 6.5%, *p* = 0.61) (Table 4).

**Table 4:** Direct-to-Catheter vs. Pre-ablation diltiazem: fibrosis

Variable	DTCA (No CCB) (*N* = 639)	Pre-ablation Diltiazem (*N* = 55)	Total (*N* = 694)	*p*-value
Fibrosis				
Baseline fibrosis (%)	18.9 ± 7.6	18.7 ± 7.6	18.9 ± 7.6	0.78
Post ablation fibrosis (%)	14.7 ± 6.5	14.3 ± 7.0	14.7 ± 6.5	0.61

**Fig. 3:**
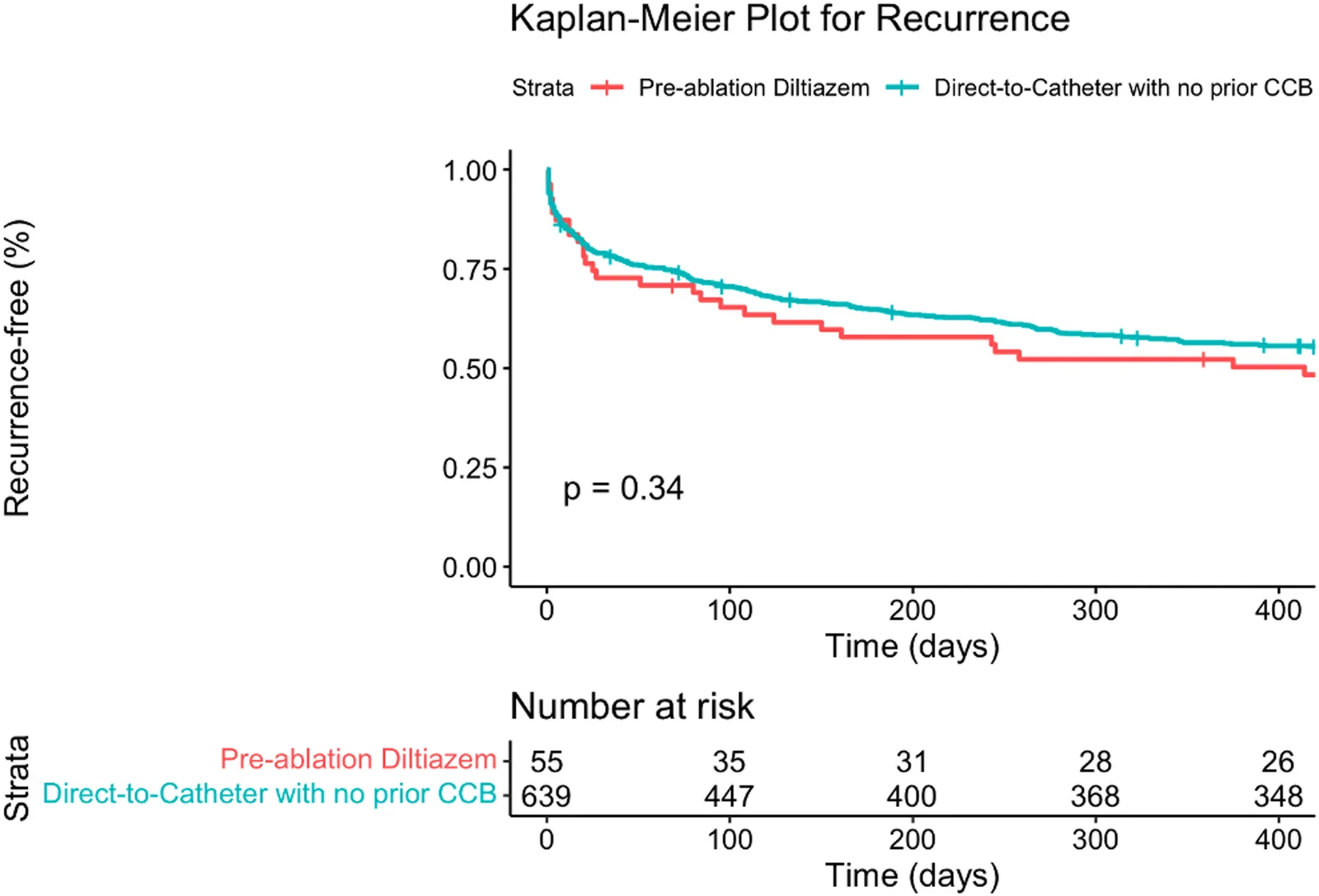
Comparison of Quality-of-Life Following Ablation (Pre-ablation Diltiazem vs Direct to Catheter Ablation with no Prior Calcium Channel Blocker). A Kaplan Meier curve comparing the risk of atrial fibrillation recurrence, in days, following catheter ablation between pre-ablation diltiazem, vs direct-to-catheter ablation with no prior calcium channel blocker (CCB). No significant difference was found in recurrence between the two groups (p = 0.34)

**Fig. 4:**
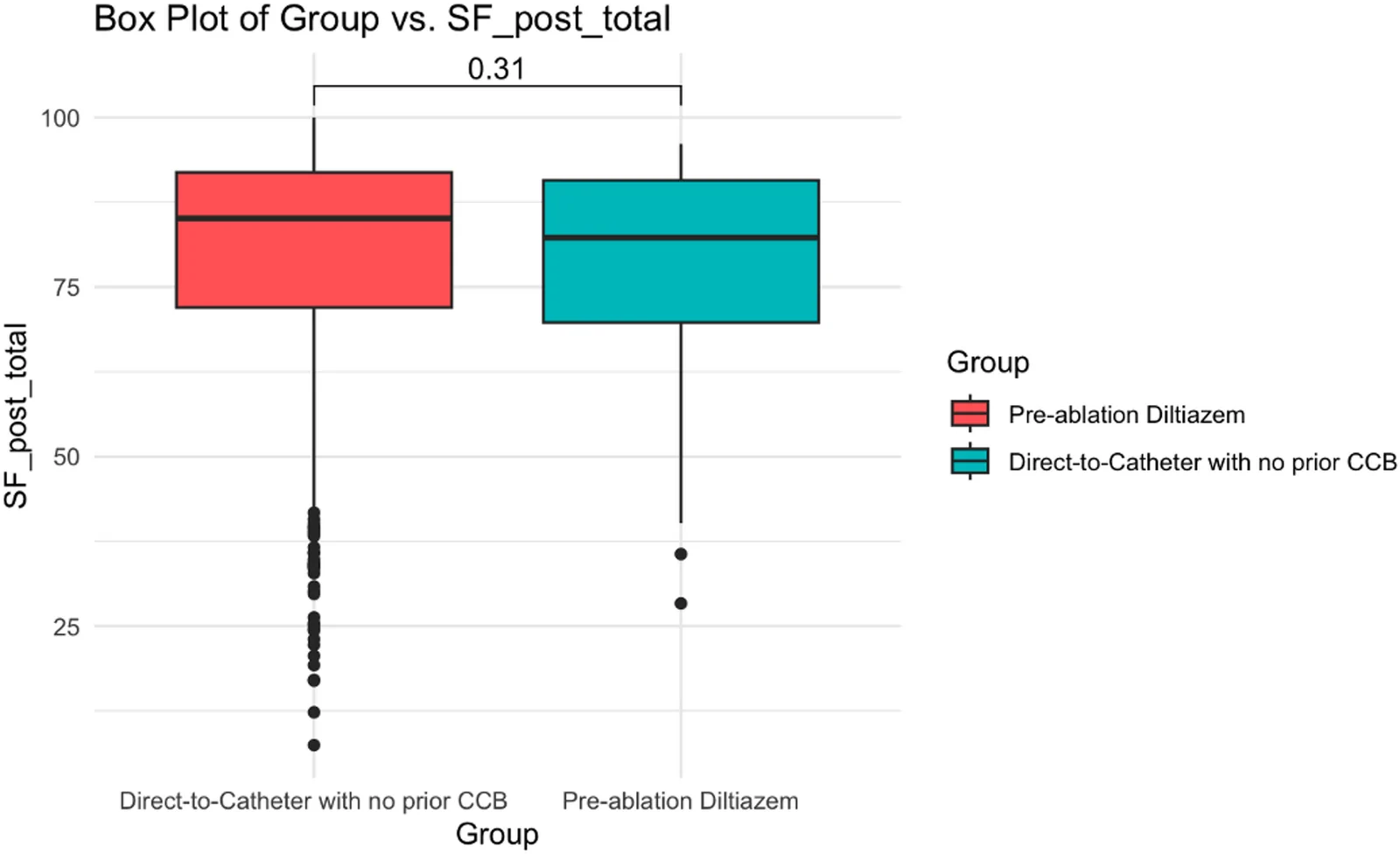
Comparison of Quality-of-Life Following Ablation (Pre-ablation Diltiazem vs Direct to Catheter Ablation with no Prior Calcium Channel Blocker). A box plot demonstrating similar quality of life, as measured by the SF 36 questionnaire, between the pre-ablation diltiazem group and the direct-to-catheter ablation without prior calcium channel blocker group (p=.31)

### Multivariable Cox regression

In both Cox Regression analysis and multivariable linear regression, the use of diltiazem before ablation had no significant effect on AF recurrence (HR = 1.12, 95%CI (0.75, 1.67), *p* = 0.57). Both male sex (HR = 0.652, 95%CI (0.49, 0.86), *p* = 0.003) and pre-ablation LA volume (HR = 1.006, 95%CI (1.003, 1.009), *p* < 0.001) emerged as statistically significant predictors. Using the same models for the other comparator, use of metoprolol prior to ablation had no statistically significant effect on AF recurrence (HR = 1.12, 95%CI (0.84, 1.49), *p* = 0.44), and pre-ablation LA volume had the most significant effect on AF recurrence (HR = 1.005, 95%CI (1.001, 1.009), *p* = 0008).

## Discussion

At 12-month follow-up, a direct-to-catheter approach appears similarly effective to pre-ablation rate control treatment with either diltiazem or metoprolol with respect to AF recurrence following ablation.

### The role of rate control agents before catheter ablation

Historically, rate control has been a foundational approach to managing AF. The AFFIRM trial established that rate control was non-inferior to rhythm control with respect to all-cause mortality in AF patients [7]. However, more recent trials, such as EAST-AFNET 4, demonstrated that early rhythm control, including ablation, significantly reduced cardiovascular events [4]. Similarly, CABANA showed that catheter ablation reduced AF recurrence and improved symptoms compared to drug therapy, although mortality benefits were not statistically significant [8]. 

Post-hoc analyses of CABANA and results from STOP-AF and EAST-AF further support ablation earlier in the disease course [4, 8, 9]. Findings from DECAAF II revealed no added value from pharmacologic rhythm control before ablation [5]. 

According to the 2023 AHA/ACC/HRS guidelines, rate control remains appropriate in minimally symptomatic patients or those unsuitable for rhythm control. However, catheter ablation holds a Class I recommendation for patients with symptomatic AF, without requiring a prior pharmacologic trial [2]. 

Our findings suggest that beta-blockers and CCBs do not meaningfully alter post-ablation outcomes thus, it could theoretically delay effective intervention. Early rhythm control, as supported by EAST-AFNET 4, is associated with fewer cardiovascular events and supports earlier use of ablation [4].

Guidelines emphasize individualized therapy based on AF burden, comorbidities, and access to procedural care. This study contributes to the growing evidence supporting DTCA as a practical and effective first-line strategy for PersAF management.

### Mechanistic insights

The studied rate control agents function differently; Beta-blockers attenuate sympathetic tone and downstream RAAS activation, while calcium-channel blockade can limit arrythmogenic intracellular calcium overload and TGF-β–mediated fibrotic signaling. These agents effectively slow ventricular response to AF, reducing the risk of tachycardia-induced cardiomyopathy, left ventricular (LV) remodeling, and improving symptoms [10–13]. Improved hemodynamic stability provided by these agents before ablation could translate to better symptom burden and overall recovery. However, despite these plausible mechanisms, our analysis did not demonstrate measurable change in fibrosis, AF recurrence, or QoL, suggesting that any such effects are likely insufficient to influence ablation outcomes in patients with advanced persAF.

It is unsure how much rate control agents can protect against LA remodeling since the LA is still subjected to the rapid depolarizations of AF. While these agents prevent electrical propagation to the ventricles, they do not prevent foci from firing. Accordingly, even with a decrease in ventricular rate, the LA may still be subject to the same remodeling forces experienced by a patient not treated with rate control. Since LA remodeling, such as dilation and low voltage, has been most consistently associated with the risk of early recurrence, it is understandable that such rate control agents may not significantly lower the risk of early recurrence following ablation [14]. AF is a progressive disease as described by the concept “AF begets AF,” in which AF episodes cause consequent arrhythmogenic changes in the LA, which promote further AF episodes [14–16]. Accordingly, symptomatic improvement with rate control agents that may delay catheter ablation may unnecessarily subject a patient to further remodeling of the LA while awaiting definitive treatment. Particularly in the PersAF population, according to our analysis of DECAAF II, the extent of LA remodeling may render the effect of rate control on recurrence after ablation negligible.

### Limitations and future directions

Our analysis of DECAAF II is limited by its retrospective nature. A lack of long-term follow-up makes it difficult to draw conclusions on differences in the long-term efficacy of ablation between groups. Additionally, given that rate control therapy in clinical practice is often initiated for specific indications, (e.g. symptomatic tachycardia, rapid ventricular response), patients requiring such therapy typically represent a clinically sicker population at baseline. Accordingly, in our study It should be noted that patients treated with rate-control therapy before ablation exhibited a higher burden of comorbidities, which, despite efforts to preform statistical adjustment, may confound any treatment effects. The relatively small diltiazem cohort limits the statistical power to detect modest differences and raises the possibility of a type II error. Larger, prospective studies with balanced exposure groups are warranted to confirm these findings. Additionally, detailed data on the timing, duration, and dosage of rate-control therapy, as well as adequacy of achieved rate control, were not available. These factors may have influenced outcomes and merit evaluation in future prospective analyses. and analysis of non-selective beta blockers and other CCBs may give a fuller picture of the differences in effects within each drug class.

Though our findings clearly support direct referral for ablation in appropriate PersAF candidates without a preceding trial of rate control, rate control remains appropriate for older, minimally symptomatic, or high-risk patients for whom ablation is deferred or contraindicated. Prospective randomized and propensity-matched studies evaluating differently aged subgroups, or with cohorts with significant comorbidity, will help refine the criteria guiding the ideal timing of ablation. Pre- and post-ablation imaging of patients with a spectrum of AF phenotypes, compared to our sole PersAF cohort, will help establish any variance in rate control effect on outcomes. Analysis of an optimal treatment length before ablation will be valuable in ensuring best practices. Lastly, use of AF-specific QoL instruments such as AFEQT or AFSS, may lead to more precise analysis of AF symptoms in future studies. Prospective studies with longer follow up period are warrented.

## Conclusion

Our analysis of PersAF patients undergoing catheter ablation found that pretreatment with metoprolol or diltiazem did not confer additional significant benefits in reducing AF recurrence, LA fibrosis, or improving Qol over a 12-month follow-up period. These results suggest that DTCA may be a reasonable alternative to pre-ablation rate control with beta-blockers or calcium channel blockers. Considering recent guideline updates, our results provide additional evidence supporting early ablation strategies and suggest that pre-ablation rate control may be an unnecessary intermediary step in patients with persistent AF. However, clinical judgment should continue to guide the initiation of rate control therapy, particularly in patients who are not immediate ablation candidates whether for clinical stabilization or operator availability.

## Data Availability

No datasets were generated or analysed during the current study.
